# Supplementary material for: Neural Inverse Design of Nanostructures (NIDN)
Source: Sci Rep. 2022 Dec 22;12:22160. doi: 10.1038/s41598-022-26312-w (PMC9780235; doi:10.1038/s41598-022-26312-w)
Supplement: Supplementary file 1 — Supplementary Information. [file 41598_2022_26312_MOESM1_ESM.pdf]

A Supplementary Information

We show the numerical comparison between the original implementation of RCWA in the Python package grcwa with the implementation in NIDN. Figure A.1 contains the details of the uniform and patterned unit cells. Figure A.2 shows the comparison in reflectance and transmittance of the unit cell from Figure A.1a and, correspondingly, Figure A.3 from Figure A.1b.

The validation of the FDTD impelementation in NIDN with respect to the original package fdtd is shown in Figure A.4 through the electric field in a TiO2 slab of 300 nm.

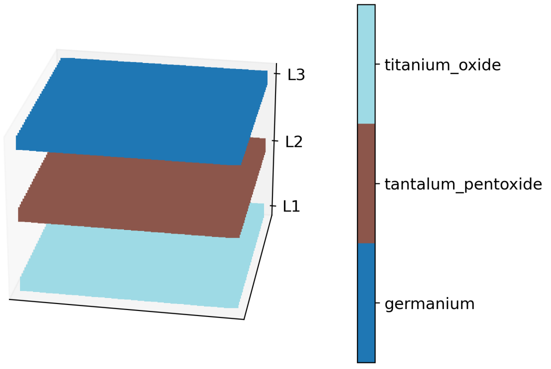

(a) Uniform Grid Test Case

(b) Patterned Grid Test Case

Figure A.1. Geometry of test cases for validation against the original implementation of RCWA in the Python module grcwa. A three-layer homogeneous material and a two-layer patterned material are investigated.

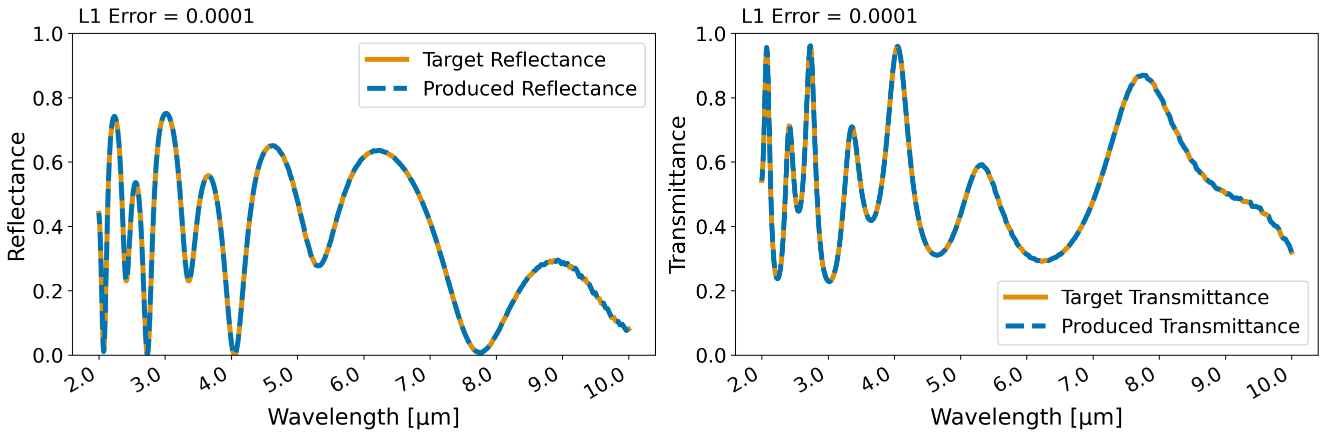

Figure A.2. A three-layer uniform material with a layer of Germanium, Titanium Dioxide (TiO_2) and Tantalum Pentoxide (Ta_2O_5), respectively. The spectral characteristics are investigated at wavelengths between 1 µm and 50 µm. The mean absolute error between GRCWA and NIDN is $7.90 \cdot 10^{-5}$. Overall, the spectra are very similar and differences are likely in the range of numerical errors.

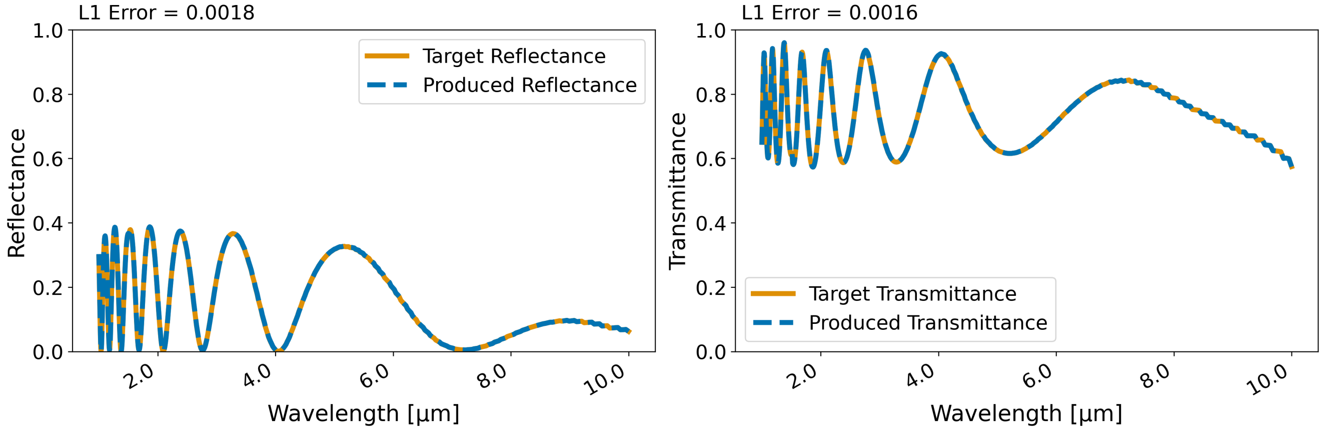

Figure A.3. The second test case is a material consisting of two patterned $0.1\,\mu m \times 0.1\,\mu m$ layers consisting of Ta_2O_5 with an embedded square and circle component of ZnO and TiO_2 in the center, respectively. A range of $1\,\mu m$ to $10\,\mu m$ is chosen. Obtained mean absolute error are between the spectra is $1.70 \cdot 10^{-3}$. Again, the obtained spectra match almost perfectly.

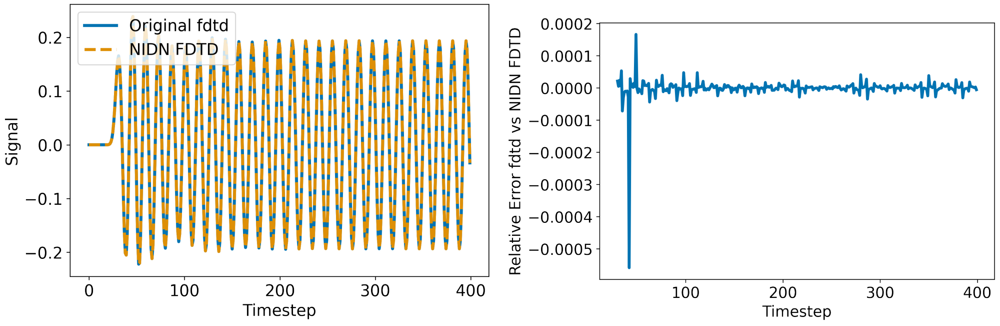

(a) fdtd vs. NIDN signal

(b) Error between fdtd and NIDN

Figure A.4. Direct comparison of a single 300 nm thick TiO_2 layer obtained with NIDN's FDTD implementation and the one from the fdtd Python module for a continuous light source at a wavelength $1\,\mu m$. The mean absolute error between the original FDTD and NIDN is $6.88 \cdot 10^{-7}$. Overall, results remain virtually identical apart from numerical errors.
